# The Expanding Scope, Inclusivity, and Integration of Music in Healthcare: Recent Developments, Research Illustration, and Future Direction

**DOI:** 10.3390/healthcare9010099

**Published:** 2021-01-19

**Authors:** Bev Foster, Sarah Pearson, Aimee Berends, Chelsea Mackinnon

**Affiliations:** 1 Room 217 Foundation, Port Perry, ON L9L 1A5, Canada; aberends@room217.ca; 2 Faculty of Social Work, Wilfrid Laurier University, Waterloo, ON N2L 3C5, Canada; spearson@wlu.ca; 3 Faculty of Health Sciences, McMaster University, Hamilton, ON L8S 4L8, Canada; mackinch@mcmaster.ca

**Keywords:** music therapy, neurologic music therapy, music care, music medicine, long-term care, social prescription, isolation, loneliness, participatory action research

## Abstract

This paper is in three sections. Section One presents a historical overview of international initiatives that have expanded the role of music in healthcare, from the initial formalization of music therapy to its more research-based rehabilitation focus to recent decades that have seen an increasing role for professional and community musicians, paraprofessional music services, music-oriented service organizations, and a very large increase in medical funding for music effects. “Music Care” is a particular and comprehensive concept promoted by the Room 217 Foundation in Canada, featuring an inclusive and integrated approach to optimizing the use of music in healthcare settings. It is part of an expanding landscape of global practices and policies where music is used to address specific issues of care. Section Two is provided as an illustration of the growing scope of the concept of using music in healthcare. It reports on a multi-year project that engaged 24 long-term care homes in conducting individualized action research projects using the fundamental approach of “Music Care”, empowering all caregivers, formal and informal, musicians and non-musicians, to use music to improve quality of life and care. Section Two presents only high-level results of the study focused on using music care to reduce resident isolation and loneliness. Section Three draws on the results from the study reported in Section Two to inform the potential and path to the future of music optimization in any healthcare setting.

## 1. Section One: The Expanding Awareness of Music in Healthcare

The first formalization of a role for music in modern healthcare was the establishment of music therapy (MT) as a profession. In the last 80 years, MT has gained recognition around the world [1] for its use of music-based interventions within the context of therapeutic relationships to support health and well-being in a variety of settings. Music therapists undergo rigorous training, completing a minimum of an undergraduate degree in music therapy, including a 1000-h clinical supervised internship, followed by successful completion of a certification exam. In many countries and regions, music therapists are registered with professional bodies e.g., the Health and Care Professions Council in the UK and the College of Psychotherapists in Ontario, Canada. Graduate level education (Master’s degree) and graduate certificates are also training options. Ongoing professional development is mandatory to maintain credentials. Music therapists work independently and on interprofessional teams. National music therapy associations are globally connected through the World Federation of Music Therapy.

While the professional practice of music therapy has become well-established in many parts of the world [2,3,4], the breadth of music use in healthcare in the twenty-first century is expanding, as will be described below. Informal (family and volunteer) and formal caregivers (paid care providers) are interested in using music in regular care practice. Trained professional and community musicians have begun to engage with healthcare settings as viable working arenas. Technology has made musical person-centered care possible. Research, policy, and advocacy for music and health is on the rise. New scopes of music and health practices are emerging. The context is changing.

This paper will landscape some of the ways that music has expanded beyond music therapy over the last 20 years and provide an example that illustrates an innovative approach to address significant healthcare issues in long-term care. 

### 1.1. The Ever-Broadening Use and Acceptance of Music in Care

#### 1.1.1. Novel Music Therapy Techniques and Applications

Music therapists have delved more deeply into specialized areas, creating valuable new techniques and applications that can be used by other medical staff. For example, neurologic music therapy (NMT) is a treatment methodology developed by Drs. Michael and Corene Thaut. From its early days in 1999 at Colorado State University to today at the University of Toronto, the Academy of Neurologic Music Therapy offers NMT training that features music-based diagnostic techniques in three functional domains: sensorimotor, speech/language, and cognition. NMT is used by music therapists and allied care professionals like physiotherapists, occupational therapists, and speech language pathologists to employ focused, music-based medical interventions. In 2015, the Oxford Handbook of Neurologic Music Therapy was second overall in the British Medical Association (BMA) Book Awards for neurology (600 entries), an unprecedented achievement for a work in music therapy. In 2019, one of the NMT techniques—rhythmic auditory stimulation—was identified as a clinical best practice in the stroke rehabilitation guidelines in Canada and the US [5].

In France, music therapist and doctor of clinical psychology, Stéphane Guétin, has developed a methodology to use music prescriptively to control pain and anxiety. His U sequence is able to synchronize music with vital signs, and can be used digitally for treatment via the MedAppCare by medical personnel [6].

In 2011, Andy Tubman, a music therapist in California, co-founded a program called SINGFIT, which uses a technology-based app to prescribe singing for residents of senior living communities. Staff members are trained in both the technology and methods of SINGFIT to deliver the program and engage residents “in musical journeys that are great for the mind, body and spirit” [7].

#### 1.1.2. Music Medicine

In the late 1970s, German medical doctors Roland Droh and Ralph Spingte began to explore ways of using music to address pain and anxiety, and attracted other doctors and academics to their newly formed group, the International Society for Music in Medicine [8]. Parallel to this organization was the International Association of Music and Medicine (IAMM) formed in 2009, predominantly made up of music therapists who were situated in medical settings. These groups eventually overlapped and joined forces as a nexus for academics, medical practitioners, neuroscientists, music therapists, and other key stakeholders in music and health. The IAMM “encourages and supports the use of music in medical contexts including research into the benefits of music and its specialized applications in healthcare” [8]. It offers a biennial conference and publishes a quarterly journal called Music and Medicine [9]. However, the terms “music in medicine,” “music and medicine,” and “music medicine” are still not clearly differentiated. The first two now basically mean the same thing: the use of music to accomplish medical purposes usually mediated by a therapeutic relationship, but recognizing that the music processing itself plays a crucial role (as in neurological music therapy), whereas, “music medicine” now mostly refers to “music as medicine”, where the expectation is that the music and sound is the therapeutic by means of its rhythmic, vibrational, or other sonic properties and how they interact at a cellular level with the human body.

For the last 30 years, the vibroacoustics field has situated itself in the music as medicine camp. Since 2012, the work of VIBRAC, a research and training center in Finland, has strengthened the scientific basis for vibroacoustic therapy, and serves as a point of contact for researchers and clinicians around the world. VIBRAC has established an international training model that reaches various fields and professions interested in integrating vibroacoustic therapy into regular care practice [10].

#### 1.1.3. Music in Health Research

Universities and colleges have begun to create institutes and centers to build critical evidence around aspects of music in healthcare. These academic centers include the McMaster Institute for Music and the Mind (McMaster University, Canada) that studies musical development of infants and children; the Sidney de Haan Research Centre (University of Kent at Folkstone, UK) that explores participation in creative arts for well-being; the Cognitive Brain Research Unit (University of Helsinki, Finland) that researches music processes and their plasticity as they relate to neural conditions; the Cambridge Institute for Music Therapy Research (Angela Ruskin University, UK) that conducts interdisciplinary research in music therapy; and the Institute of Music Physiology and Musicians’ Medicine (Hanover University in Germany) that researches conditions of performing musicians [11,12,13,14,15].

Research funding is an indicator that music and health is becoming a social priority. In 2016 in the US, the Sound Health initiative was launched as a partnership between the Kennedy Center and the National Institute of Health (NIH), the largest funder of biomedical research in the world. The importance of this research examining the evidence for and mechanisms of the effects of music on health conditions and the investment cannot be overstated. In 2019, the NIH granted 20 million USD to fund the first set of projects more narrowly focused on questions emerging from neuroscientists. The second round of funding focused on exploring how music impacts normal health and development, rigorous studies of music interventions to treat disease symptoms, and promoting the understanding of basic mechanisms through which music is processed by the brain and body [16]. Also important is the profile of the working group overseeing the Music and Health funding program—it is not a “music therapy” group, but rather a broad-ranging medical expert group [17].

#### 1.1.4. Musicians in Healthcare

As research and practice in music and health continues to expand, an increasing variety of non-music therapists and non-medical personnel are using music in care settings. Professional musicians are bringing their musical practice into healthcare contexts to engage residents and patients in aesthetic and creative experiences. Programs in the UK include Music in Hospitals and Care [18] and Live Music Now [19]. The Music for Life program [20] at Wigmore Hall in the UK trains participatory artists to work in residential healthcare settings with persons living with dementia as co-creators of music.

In 1999, Musique et Santé, a not-for-profit organization in France, established an apprentice program for professional healthcare musicians trained to work alongside care and medical staff in traditional healthcare spaces with cultural rather than clinical goals, engaging and interacting closely with patients at bedside. This practice has now extended into Europe and other parts of the world [21].

Community musicians grew out of a socio-political movement in the UK in the 1960s. As a global practice, it evolved from a grassroots endeavor to a field of study in higher education. According to Higgins and Willingham, community music practice has widened its perspectives and application to various health and social care settings [22]. For example, Converge is a partnership between York St. John University and a national health service trust in the UK that contributes to mental health. Music is the core of Converge, offering courses to persons living with mental illness in composition and songwriting, as well as a weekly choir called Communitas. Outcomes of Converge include an increased sense of belonging and social integration [22]. The Oakdale Prison Choir in Iowa is focused on social cohesion. Founder and community musician, Dr. Mary Cohen, aims to meet the needs of the incarcerated men, which she identifies as “having few artistic expressive outlets”, with the women and men from the outside who all come together to sing [22].

Musicians who are not trained music therapists but with niche music training have gained momentum in healthcare over the past 20 years. For example, harp therapy refers to several practices that use the harp as a therapeutic agent in home and clinical settings for specific outcomes, like pain reduction, end-of-life care, and sensory stimulation [23]. In 2003, the founders of several international harp therapy programs created the National Standards Board for Therapeutic Musicians (NSBTM), which was incorporated in 2007 and established curriculum, an accreditation process, and a code of ethics [24]. Member affiliates include Bedside Harp [25], the Clinical Musician Certification Program (offered by Harp for Healing, LLC) [26], the International Harp Therapy Program [27], and the Music for Healing and Transition Program [28].

#### 1.1.5. Equipping Caregivers

While scopes of musical practices are expanding in healthcare, so grows an interest in equipping formal and informal caregivers with musical skills and resources. In 2002, Eva Göttell and her colleagues in Sweden began to report on relevant research they were doing with formal caregivers in care homes who did not necessarily have any formal training in music. By adding singing and music to formal care routines for persons living with dementia, there was an increase in mutuality of interaction. Specifically, staff noted that the patients had straightened posture, stronger movements, and increased awareness of themselves and their environment. Positive emotions were enhanced, and aggressiveness diminished [29,30,31,32].

In 2009, the Room 217 Foundation [33] began in Canada. Room 217’s focus is on supporting caregivers with designed music products, education, and training that can be integrated into any caregiver’s practice, whether the individual perceives themselves to be musical or not. Room 217’s educational opportunities are unique in that they cater to both formal and informal caregivers—any interested individual who might want to learn about evidence-based uses of music. Most notably, the Room 217 Music Care Conferences provide a platform for rich learning and networking opportunities between informal and formal care providers with regards to the use of music in care.

#### 1.1.6. Music-Based Programming

Several music-based programs have become mainstream in healthcare communities and communities at large. In 2014, the film Alive Inside [34] popularized the notion that personalized music could dramatically improve quality of life for residents in long-term care and nursing homes. In a visual way, the film followed the work of New Yorker Dan Cohen and the organization he founded in 2006, called Music and Memory [35]. A former social worker, Cohen began to offer personalized music playlists on iPods to residents. Since that time, more than 5000 nursing homes worldwide have engaged in Music and Memory certification. A few years later in 2013, the Scottish-based charity Playlist for Life began its work in the community to improve quality of life for people living with dementia and their caregivers. This group has created accessible programming in partnership with the BBC, as well as downloadable tools that formal and informal caregivers can use with impressive community reach [36]. Another popular music-based program is Singing for the Brain, a standardized service delivered through the UK Alzheimer’s Society since 2003. Trained program leaders offer weekly singing sessions in community settings. The model incorporates “social interaction, peer support, engagement and active participation to improve quality of life, communication and social engagement and to enhance the relationship between the person with dementia and the caregiver” [37].

#### 1.1.7. Technology

Technology as a means of music delivery in healthcare has substantially expanded. The virtual music instrument has been developed for multiple applications e.g., children with special needs at Holland Bloorview Hospital in Toronto [38]. An interface provides an opportunity for children who may not be able to hold or manipulate an instrument to do music by translating physical movements into music. Dr. Timothy K. Shih at the MINE lab at the National Central University in Taiwan is taking the virtual music instrument a step further by using 3D finger gesture tracking and recognition [39]. Another established music technology platform is Soundbeam, which was developed over the past three decades in the UK. Soundbeam has been supporting seniors and children with special needs with multi-media experiences, combining sensor technology and visuals to translate body movement into music and sound [40].

Virtual reality and artificial intelligence applications are being developed for delivering music into healthcare contexts. Currently, an American start-up called n*gram health has begun to use virtual reality to deliver virtual and augmented reality applications for seniors [41]. A group out of Toronto called LUCID is developing what they call musical artificial intelligence experiences to specifically target issues in mental health [42].

#### 1.1.8. Policy and Advocacy

One of the most significant growth areas is the World Health Organization’s developing agenda for arts in health. In November 2019, the World Health Organization presented the largest evidence report ever published on arts and health, which gives an overall picture of the role the arts play in supporting health globally [43]. Dr. Daisy Fancourt writes of this scoping review:

The findings are structured into two sections. The first focuses on prevention and promotion: how arts can affect social determinants of health, support child development, encourage health-promoting behaviors, help to prevent the development of mental and physical illness, and support caregiving. The second section focuses on how the arts can support the management and treatment of mental illness, neurodevelopmental and neurological disorders, and non-communicable diseases, as well as support the delivery of acute care and palliative care. The findings are illustrated with a selection of case studies from across the WHO European region and presented alongside a series of policy options for how to support the development practice and research internationally [44].

A world leader in national policy around music and health, the UK formed an All-Party Parliamentary Group on Arts, Health and Wellbeing (APPGAHW) in 2014. The APPGAHW aims to improve awareness of the benefits that the arts can bring to health and well-being. This group conducted a three-year national inquiry and made recommendations to improve policy and practice to the government [45]. A group that has played a large role in music advocacy for dementia care in the UK is the Utley Foundation [46]. This private family charitable trust funded a commission on music and dementia, researching and recommending best practices in the UK [47], and led the national campaign Music for Dementia 2020. Although the campaign was interrupted by the pandemic, a comprehensive and resourceful website has been created for caregivers including a national mapping of dementia community programs [48].

#### 1.1.9. Social Prescription

One of the most recent developments in music and healthcare in Japan and the UK is the advent of social prescription [49,50,51,52]. Social prescription, also known as community referral, is a way that general practitioners, nurses, and other care professionals refer people to a spectrum of local non-clinical services. The goal of social prescription is to address people’s needs in a holistic way and support individuals to take greater control in their own health and well-being. Music is one of many activities that can be prescribed e.g., singing in a choir or taking lessons. The integration of the health system and local sources of support is fundamental to this approach working. The potential to expand music within the social prescription framework is ripe.

#### 1.1.10. COVID-19 Pandemic

Most recently and without forewarning, the world has witnessed music’s powerful connection to societal health during a global pandemic. Countries have offered musical tributes to frontline care workers. On a local level, neighborhood concerts demonstrated how music mitigates isolation and keeps mental health in check. For example, Italy turned to music during COVID “to create a nationwide concert to lift our spirits and make a daily appointment to be social” [53].

#### 1.1.11. Summary Section One

This section has shown the growing interest in music in healthcare in the past two decades. Previously, music therapy as a profession had established itself as a practice, but research to demonstrate its efficacy has in general been on the weak side [54]. Most of the initiatives of the past decades, perhaps with the exception of neurologic music therapy and recent efforts in music medicine, have either very sparse or just emerging research. It is clear that for the medical healthcare field to integrate music into an expanding scope of practice, substantial research efforts, of the sort very recently supported by the NIH in the US, will be required. At present, the practice of music in healthcare is developing ahead of a full understanding of the scientific foundation, especially of mechanisms of action, required for long-term sustainability.

## 2. Section Two: The Expanding Scope Illustrated

### 2.1. Approach

#### 2.1.1. The Music Care Approach

One current concept that is gaining traction is the music care approach [55], which promotes the use of sound and music to meet challenges of care. It is an approach that values and engages the musical efforts of all caregivers, not just those who are musically trained. It is an approach developed and promoted by the Room 217 Foundation and builds on the inclusion of the 10 Domains of Music Care (see Appendix A). It represents many of the developments related to music and healthcare of the past two decades. The music care approach seeks to empower all caregivers, not just trained musicians, formal and informal, to use music to improve quality of life and care. The goal of this approach is the integration of music into caring relationships, care tasks, personal care plans, and within caring communities. In short, the music care approach is a framework that a variety of stakeholders can use to determine a musical solution to address a care-related problem.

Over the past four years, the Room 217 Foundation has facilitated action research in 27 long-term care (LTC) homes in the greater Toronto area to implement the music care approach. A pilot study was done first in 2017 in three long-term care homes using a music care action research project (MCAP) to address the experience of resident isolation and loneliness.

When someone transitions into LTC, no matter their age, the move is often accompanied by isolation, loneliness, or both [56,57,58,59]. Social isolation and loneliness have been identified as risk factors for physical and mental health problems for older adults [60]. Often used interchangeably, they are distinct. Social isolation refers to an objective measure of the number of social contacts and interactions one has, whereas loneliness is a subjective experience or feeling and is perceived negatively [61]. Isolation and loneliness have always been significant challenges in LTC, made more acute and public during the current COVID-19 pandemic.

A key output from the pilot study was the development of the integrated model of music care (IMMC)—a concept of the necessary factors to integrate music into standard healthcare. Foundational to the IMMC is training and information, understanding that music can have both beneficial and adverse effects on a person’s well-being [62]. The goal of training was to increase caregiver confidence and skill to use music in some capacity, regardless of prior musical training. Building on that knowledge, caregivers determined a purposeful intention to use music to reduce the experiences of resident isolation or loneliness. With the assumption that an action research strategy would lead to the necessary engagement and investment in solving a situated locally perceived problem, each participating site developed their own unique action plan. An action plan was developed and implemented through a measurable program, care task, or therapeutic relationship. Changes were tracked by process and progress evaluation tools. Music care integration in LTC happened when music was assimilated into the care environment as a means of change, when most care providers saw music as a viable option to address human challenges, and when most care providers were able to follow a process of intentionally introducing music into the care setting. Using the IMMC as a conceptual framework, a two-year study was conducted in 24 LTC homes using the music care approach.

Research data should inform any expansion of the role of music in healthcare. Unfortunately, many of the initiatives employing music in health contexts of the past two decades have little to no research foundation. The Room 217 Foundation that has developed and promotes the music care approach expects that the implementation of music care in LTC homes will be informed by research data, so systematic data collection related both to the process of implementation and to the results of the implementation contribute to the knowledge base supporting and developing the music care approach. One of the most well-suited research methodologies for understanding the process of an implementation and the effects it has is participatory action research. This research exemplifies how the scope of music in healthcare is expanding in LTC settings to combat the prevalent challenges of resident isolation and loneliness.

#### 2.1.2. The Study: The Effect of Music Care on Loneliness and Isolation in Long-Term Care

A study was designed to explore two things simultaneously: (1) how can an LTC facility effectively implement the Room 217 music care approach? and (2) what effect would a locally determined music care project have on residents’ experiences of loneliness and isolation? To answer these questions, essentially, 24 case studies of LTC homes were conducted. The means of the implementation was the facilitation of a locally designed action research project. Cross-case analysis was done to determine any effect from the diverse projects on loneliness or isolation.

### 2.2. Purpose

The purpose of this study was to determine the effects of a music care action project (MCAP) on the isolation and loneliness experienced by residents in LTC homes in Ontario, Canada. Research questions included:

1. Does a situational implementation of an integrated model of music care change LTC residents’ experience, especially as it pertains to isolation and loneliness?
2. How do LTC homes begin to integrate music on a day-to-day basis into the community culture?
3. What are the factors that contribute to a successful MCAP?

### 2.3. Methodology

**Figure 1:**
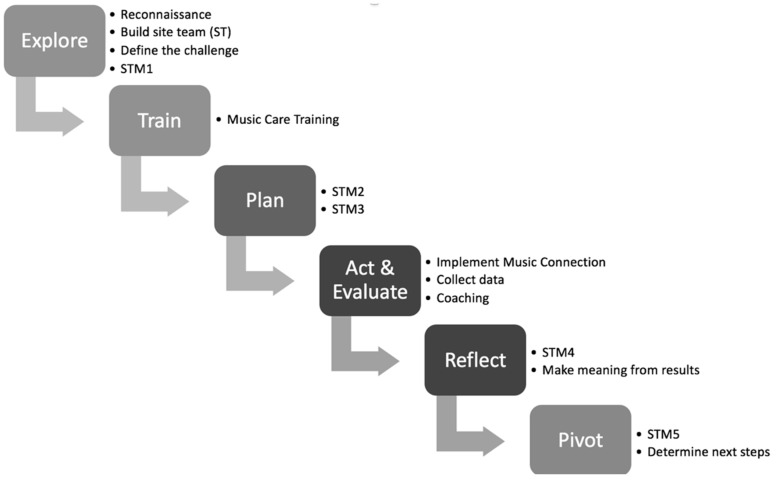
Six-step action research design (STM—site team meeting).

The basic methodology used was a six-step participatory action research (PAR) design (see Figure 1). Action research as a methodology emerged from a context of social action in the 1940s with the intent of formalizing the process of “trying something” to change practice within an organization. With classic action research, an outsider can initiate action within an organization. With PAR, more responsibility rests on personnel within the organization to take initiative to define and conduct the action to be taken. A feature of action research is the use of multiple data sources from which conclusions of efficacy can be reached. PAR was chosen because it aims to improve community challenges and involves the people who can take action to make the improvements. Action research puts the onus on the community to put steps in place to solve a systemic problem (in this case, reducing resident isolation and loneliness), evaluate efforts, and make adaptations along the way. Steps are more likely to be systemically maintained, as research is not done to, but with key community players who are decision-makers and agents in sustainability efforts [63,64]. Because a PAR approach was used in multiple LTC homes, the nature of the actual intervention varied with each home. The result was a series of case studies using PAR focused on loneliness and isolation. Because of common outcome measures, cross-case data could be examined, and overall effects could be observed.

The outside researcher facilitators motivated the PAR process by having five meetings with each site team, following the steps of the PAR design. Site teams are indicated as STM1–5 in Figure 1. Key outputs of the process were exploring (reconnaissance between investigators and LTC site team, defining the issues), training (baseline music care training, including sound and music theory with experientials and strategies), planning (choosing a strategic goal within the music care delivery framework, determining evaluation tools and recruitment criteria, establishing steps and timelines, and assigning responsibilities), acting and evaluating (implementing the music care initiative plan, collecting and analyzing the qualitative and quantitative data), reflecting (LTC site team/community making meaning of the results), and pivoting (celebrating the music care initiative or intervention and results, determining next steps).

### 2.4. Design

The study took place in 24 LTC homes recruited from three cities in the greater Toronto area. Room 217 recruited these homes and had a memo of understanding with each participating home (see Appendix B). Study participants within each LTC home were selected and recruited by local site team members in each LTC home based on who they thought would benefit the most from this research project in terms of demonstrated loneliness or isolation i.e., not participating, staying in the bathroom for long periods of time, etc. Informed consent was obtained by Room 217 with the help of site staff from each participant (see Appendix C) or from their power of attorney. Ethics review and approval was provided by the Veritas Independent Review Board.

A total of 265 residents were enrolled as participants in action projects in these 21 data-reporting LTC homes. Fifty-six percent of participants were female and 44% were male, with a mean age of 78.5 years. Fifty-two percent of participants were living with moderate to severe cognitive impairment (CPS score of 3+).

While the COVID-19 pandemic did not impact the delivery of the action project or the subsequent continuation of music care as a regular practice within the LTC home, it did impact post-data collection at three participating sites. This is because site team members were redeployed or their roles were redefined to support urgent pandemic-related tasks and activities. Legislation implemented to protect residents prevented data collectors from entering the home to interview residents after the study was complete. Consequently, data was obtained from 21 LTC homes.

The entire PAR process took between six to eight months to complete in each home. A site-specific MCAP was implemented for two months at each LTC site, and focused either on the outcome of isolation or of loneliness. Note: Each LTC home decided on their own specific action plan. The action research interventions implemented in the participating LTC homes included: 1:1 music visits, choirs or singing sessions, making music care kits, music clubs, bursts of spontaneous musicking, handbell choirs, care plan integration, intentional mealtime music, and culturally-specific music. In each case, it was the intention of staff to address the need of isolation and loneliness with music.

Four projects were individually based, eight projects were group based, and 12 projects involved both individual and group elements. Individually based projects are when the majority of music care is experienced in a 1:1 context; group-based projects happen when the majority of the music care is experienced in the context of a group.

Pre- and post-isolation and loneliness measures were evaluated using one of two validated tools. The Friendship Scale [65] measured isolation, and the Jong–Gierveld Loneliness Scale [66,67] measured loneliness. Data from the Resident Assessment Instrument-Minimum Data Set (RAI-MDS) 2.0 [68], the standard assessment tool in Ontario LTC, was also collected and used by RAI-MDS coordinators at each site at the end of the two-month action research project. Qualitative interviews with residents and staff were integrated into the PAR process at each LTC home. NVivo [69], a qualitative data analysis software, was used for theme analysis in the scaled study.

Noteworthy in the expanding role of music in healthcare is the shift from only certified music therapists using music in care to many healthcare workers participating in the use of music in care. There were many players involved in completing this implementation of music in care. Key roles and responsibilities for participatory action research are described in Table 1.

Credentialed professional music therapists were significant leaders in this study and were used in three roles: as program facilitators (see Table 1), music care training instructors, and staff experts. As instructors, music therapists delivered the two-day, 14-h education program to the LTC home site team. The MCAP was not dependent on the presence of a music therapist on staff at the LTC home. However, in several of the LTC homes, a music therapist was on staff and provided valuable expertise and support for the MCAPs.

### 2.5. Results

#### 2.5.1. Quantitative

Pre- and post-intervention Resident Assessment Instrument-Minimum Data Set (RAI-MDS) data were collected. This included five assessment domains: the Cognitive Performance Scale (CPS), the Aggressive Behavior Scale (ABS), the Index of Social Engagement (ISE), and the Depression Rating Scale (DRS); when possible, homes also provided data for the Changes in Health, End-Stage Disease, Signs, and Symptoms Scale (CHESS).

Five LTC homes saw an average positive change in RAI-MDS CPS scores, indicating that the implementation of a music care initiative positively impacted the cognitive function of participating residents. Seven LTC homes experienced positive changes in ABS score. Six homes demonstrated increased ISE scores. Nine of the homes reported decreased DRS scores (i.e., decreased depression) experienced by program participants. Four homes were able to report on CHESS, and one home saw an average positive change in this score over the course of the action project.

The majority of sites (67%) collected data on resident isolation using the Friendship Scale. The remaining 33% used the Loneliness Scale to collect data on resident loneliness. Each site determined that choice. On the validated isolation and loneliness tools, not every home documented average positive changes. Contributing factors include resident cognitive impairment, which could impact the ability to respond to a verbal questionnaire, the fit of resident participants to the project, translation of validated scales, and external events, such as outbreaks, the COVID-19 pandemic, and personal health changes. 

A responder on the Friendship Scale showed an increase in average post-project scores, indicating a decrease in the experience of resident isolation. A responder on the Loneliness Scale displayed a decrease in average post-project scores, indicating a decrease in experience of resident loneliness.

Additional residents may benefit from the ripple effects of music care action projects. The ripple effect occurs when music care (the empowering of all caregivers, formal and informal, to use music to improve quality of life and care) is expanded and spreads beyond the scope of the project and is an indicator of music care integration. The ripple effect manifested in three ways:

1. More residents were involved in the chosen music care initiative.
2. Additional music care initiatives were created.
3. More LTC challenges were impacted in addition to isolation and loneliness.

In one LTC home, a majority of residents had a mental health diagnosis, and tended to be marginalized and disenfranchised, and were often homeless prior to living in LTC. The youngest resident was 28. These unique demographics contributed to the experience of isolation and loneliness at the home. Music at Mealtimes was the name of this site’s MCAP. Playlists using resident-requested songs were created. Speakers and MP3 players were purchased for each dining room, and music was played during all three meals each day for eight weeks. There was a 2.61 increase in average Friendship Scale scores, which means self-perceived isolation decreased for project participants during this time period. The site team decided to collect additional data of ripple effects resulting from other positive changes because of the MCAP. For example, the LTC home average RAI-MDS aggressive behavior score decreased. The number of residents with pressure ulcers, associated with undernutrition and weight loss, decreased by 50%. Fifty-six percent of participating residents gained weight. Staff explained that residents were focused on enjoying the music and eating their food rather than watching or engaging in altercations. The amount of food being returned to the kitchen after each meal decreased from 30% to 5%. It was reported that one resident participant went from eating 25% of her meal to 50–100% when the music was on.

#### 2.5.2. Qualitative

Music care training designed for staff, volunteers, and family was integral to the MCAP. Helping caregivers feel confident to use music effectively and responsibly was the objective. One family member who was a site team participant shared, “Before I began the music care training, I thought I knew a lot about the benefits of music. I had been singing to my mom for years, who has advanced Alzheimer’s disease, and have seen how beneficial the music was. I was amazed at how much I did not know and to learn how powerful a tool music is and the many ways it can be used. For example, how music and humming can have a calming effect; how ascending and descending melodies have different effects and how music can evoke memories and reduce depression and loneliness.”

Several sites reported that, during the initial grim days of COVID-19, the music care training that was conducted for the MCAP had prepared their frontline workers to use music as an important connection with residents and their separated family members. At one site, care workers who had music care training in MCAP pivoted to help family members create playlists for their loved one to listen to.

A significant part of the research methodology was to conduct 5–15-min (dependent on cognitive capacity) in-person or phone interviews with some of the residents and staff at some of the homes to gain more understanding of their thoughts, feelings, and insights into the music care experience (see Appendix D). Thirty-two individuals from 15 different LTC homes were interviewed: 11 residents and 21 staff members of varying positions. Researchers selected nine content-rich interviews that served as initial observational data to find preliminary code structure and themes. An analysis team was formed of seven individuals with varying degrees of knowledge of the MCAPs to ensure triangulation of the results. The team categorized, rationalized, and interpreted seven themes or core concepts (describing the program and benefits) that emerged from recorded and transcribed interviews as follows:

1. Limited Resources (informants repeatedly pointed to a lack of resources to expand music activities)
2. Distinct Experiences (interviewees valued the uniqueness of the music experiences created by the project)
3. Life Enrichment (personal changes in behavior and mood occurred through music care)
4. Dynamic Relationships (symbiosis between the impact of relationships and how music care was delivered)
5. Program Flexibility (MCAPs were adaptable)
6. Potential Continuity (MCAPs had the potential to become sustained and integrated for longer-term impact)
7. Enhanced Socialization (MCAPs created a space where residents felt connected to each other and to staff)

The multiple data types and sources in this study give a richer understanding of numeric results, providing a more personal narrative and background about the effects of music on resident experiences of isolation and loneliness and what is needed for more successful music care integration from the staff perspective. In this way, quantitative and qualitative results are complementary.

### 2.6. Summary Section Two

The action research project reported had two facets: (1) a study of the process of the implementation of the music care approach in 24 LTC homes and (2) a study of the effect of music care activities on loneliness and isolation among the participants. Results of the first facet are primarily reported in Section Three, and the principal limitation for a general understanding of the role of music in healthcare was the fact that the MCAPs were conducted only in LTC homes. It cannot be known from this study whether MCAPs would have similar or different results in other healthcare contexts, like hospices, or hospitals. The second facet was reliable and important at one level: it showed that the music care approach, even with diverse activities or projects, resulted in positive effects. However, at the level of determining the activity effect rather than the music care approach, it had serious limitations. If viewing this level of the study as a clinical trial, which it was not within the context of an action research study, it fell short on crucial dimensions due to the lack of standardization, control, common measures, or comparison groups.

Future research needs to study the process of implementing music care in multiple healthcare settings in other locations in Canada, where healthcare systems and parameters may differ, or in other countries, which may have other parameters altogether. Future research on the effect of music care activities on isolation and loneliness needs to be done as rigorous clinical trials. Future research also needs to explore the effect of music initiatives in LTC on other outcomes.

## 3. Section Three: The Potential and Path to the Future of Music in Healthcare

A review of section one of this paper shows that the many initiatives over the past decades fit into one or more of the 10 domains (Appendix A) described by Foster et al. [56] and promoted by the Room 217 Foundation [33]. Predominant in the programs and initiatives described in section one is the outside-in or specialized delivery model used—the medical organization, whether LTC or hospital, does not need to have its general caregivers take ownership and participate. Furthermore, the presence of one or even several of the initiatives or services described in section one does not rise to the potential optimization of the effect that music can have in healthcare. To reach the potential of music in healthcare, all three categories of domains (Appendix A) must be employed: (1) an intentional music-rich environment; (2) professionally informed care with music; and (3) participation in knowledge development and translation. However, crucial to this future direction are two things: (1) the internal ownership and sustainable commitment to a music care program and (2) a full understanding and commitment to the concept of music care. The music care concept is to empower all caregivers, formal and informal, to use music to improve quality of life and care. The goal of music care is the integration of music into caring relationships, care tasks, personal care plans, and within caring communities. In short, the music care concept is a framework that a variety of stakeholders can use to determine a musical solution to address a care-related problem.

The study in section two of this paper is an illustration of a method to systematically introduce and develop the internal ownership and the sustainable commitment to music care. The study reported did not develop the full potential of all three domain categories, but introduced the crucial aspects on which such potential could be built: music training of general staff, an organizing team, an initiative to use music programming, participation in data collection for effect monitoring, and, in places, opportunities for music therapists to work alongside general staff. What follows is an examination of how the study reported in section two can inform future efforts by organizations and initiatives around the world to reach the potential of music in healthcare.

First, the study is an example of how research based on the music care approach is actually reaching into a healthcare setting—LTC, one that doesn’t typically focus on research – and engaging staff, residents, family members, and volunteers to think differently about music’s possibilities as a meaningful solution to challenges. The study compels us to consider aspects of the music care approach that could be expanded into more healthcare settings beyond LTC. Engaging the stakeholders of the institution in a process of systematic inquiry develops ownership and sustainability.

Second, the action plan used in the MCAP integrates all of the music care efforts. Local LTC homes synthesized and applied information about isolation and loneliness, music care delivery domains, and strategies learned in training to their specific site. Each action plan was self-designed and carried out by the representational site team, which resulted in a keen sense of shared responsibility for implementation and accountability. The plan was supported by a music care expert, such as a community musician or music therapist. At the end of one cycle of the MCAP, the pivot step in the PAR methodology allowed for the site team to repeat or adapt the action plan to meet the same outcome, or to address a different resident or community need. In some cases, the next MCAP included expanding into another music dimension i.e., hiring a music therapist or training the physical therapist on staff in NMT. The site-responsive action plan becomes a critical component of integrating, sustaining, and optimizing music in all healthcare contexts.

Fourth, playlists delivered on MP3 players, tablets, or computers were only minimally used in the MCAP study illustration. In light of the innovative way music technology is being leveraged globally in healthcare (see Section One), there may be a gap in how it is being used in LTC compared to other settings. Expanding the use of technology to deliver music in LTC will need to become a priority for providers. It may be that in the case of the MCAP study, training did not provide enough technology-based project ideas, or that the staff was reticent to use technology for delivery. It may also be institutional policy matters, since in some instances, staff were not permitted to use personal devices at work, so technological applications were limited.

Third, evaluation is a core component of any effort to measure the impact of music in healthcare. The MCAP study had several evaluation activities including (1) an initial assessment of what was happening around the use of music in the LTC home prior to the beginning the project; (2) pre- and post-data collection around the action plan; (3) site team collective reflection through collaborative analysis, interpretation, and judgments of site-specific results; (4) reporting results prepared by facilitators, including a customized report; and (5) follow-up with project leaders to determine the ongoing effectiveness and integration of music care at each participating site. Collecting evidence of music’s effects, analyzing, and making meaning of it is a significant part of efforts to integrate music into healthcare. When this approach extends into other health contexts, evaluation, whether by checklist or by more rigorous means, must be built in to determine whether changes occur.

Fifth, while quantitative and qualitative results from MCAPs describe outcomes as they pertain to isolation and loneliness, understanding what makes the projects work from an operational perspective may be just as important if music is to be expanded into healthcare settings as an integrated approach. As part of the report to each LTC site, enablers and barriers of success were explicitly stated and explained in the context of that site. They have cumulatively been grouped into categories, named as a factor (in this way, the factor could be an enabler or barrier), and given a diagnostic question to help determine readiness factors of success in music care delivery (Table 2).

From this study, the collection of music care delivery readiness factors for success gathered during MCAP evaluation may help predict the success of future projects and address any gaps prior to starting a MCAP in order to ensure readiness. While this tool was developed within the long-term care setting, it could be applicable to other care settings where MCAPs take place.

## 4. Recommendations and Conclusions

The number and range of initiatives in music and healthcare reviewed in section one and the example of an implementation effort for a comprehensive use of music in health settings in section two demonstrate the potential of an expanding role for music in healthcare. Particularly, the music care approach, with its focus on inclusion and integration, is an example of how music can be expanded for operational sustainability. Music becomes a top-of-mind response amongst a care team, whether a music therapist is present or not, to solve a community challenge. The PAR process creates the methodology for the shared response, while the MCAP becomes a tangible and accountable intentional effort towards achieving an agreed upon outcome.

What can we learn from the music care approach illustrated in section two that can be expanded upon by initiatives around the world?

1. The music care approach could become a best practice model for several reasons. (a) It is meant to be integrated into what already exists, specifically personal care plans, care tasks, and programming, into the ethos of the care community. (b) The standardized adapted PAR process integrates outcome measures as the motivation for community-wide adoption and practice. (c) Evaluation is adaptable according to the outcome being measured. (d) It is manualized, scalable, and suitable for widespread adoption.

2. A standard of music care will be needed in order to maintain quality and scope of practice ethics. Guidelines need to be articulated, including competencies, certifications, integration, results, etc. In this standard of care, there must be room and direction for all stakeholders, including care staff, volunteers, family members, patients/residents/clients, and community groups.

3. A music care certification model could be introduced into LTC and potentially other healthcare settings that accounts for various dimensions of music care delivery (see Appendix A). The planned and sustained use of music care domains are observed within healthcare organizations by individuals, training institutions, and professional organizations, and accredited by a third party.

4. A baseline music care training needs to be provided for students training to be formal caregivers in healthcare. Curriculum in colleges and universities, at the very least, needs to provide an evidence-based understanding of the beneficial and adverse effects of music, a landscape of music care delivery best practices and approaches and how to access them, and strategies and interventions that can be integrated and safely used.

5. Music therapists are positioned for leadership in the expanding landscape, and must see the whole, not just their part of the picture. Music therapy training will need to include curriculum on teamwork, working inter-professionally, systems leadership, and survey courses on other music health delivery practices.

6. Technology must become a strategic driver in music care delivery. During COVID-19, music care providers pivoted to video conferencing platforms to provide continuity of care. Frontline care workers used tablets and phones in unprecedented ways. It may be that new frontiers have opened for technology-assisted music delivery in LTC, making it more acceptable and assessible.

The expanding awareness and scope of music in healthcare over the past twenty years has been exponential. While music therapy has gained global acceptance, more players and practices have emerged and pushed the boundaries of thinking, practice, experience, and interested stakeholders in music and health. The pandemic has shone light on the urgency for greater quality of life approaches in LTC in Canada: 80% of COVID-19 deaths have occurred thus far in LTC [70]. The need for music has never been greater. Music care is an actionable and accessible approach that can be used by all caregivers, formal and informal. With an emphasis on action, training, and evaluation, music care can be integrated into daily life in LTC and potentially other care settings in an operationally sustainable way. The music care approach as a potential best practice and pathway to a standard of musical care demonstrates the potential that exists within music and health when creativity and collaboration are at the core of problem solving.

## Figures and Tables

**Table 1:** Study key roles and responsibilities.

Role	Description
Program Facilitators	Worked closely with each participating LTC home in terms of the MCP program. Coached sites through musical techniques; facilitated meetings and meaning making; consulted with site team leader throughout the program.
Research Assistants	Collected quantitative and qualitative data, assisted in data analysis and reporting.
Site Team Leaders	Oversaw the MCP program and associated program evaluation within their individual LTC home.
Site Team	Participated in five site team meetings, rolled out music care initiative at their individual LTC home.

**Table 2:** Music care delivery readiness factors.

Category	Factors	Readiness Question
Integration—What does it take to incorporate music care into the setting?	Strong leadership	Is there decisive oversight and advocacy for music care?
	Resident engagement	Are residents involved in decision-making?
	Music therapy	Is there a music therapist on staff?
	Perceptions of music and care	Is music perceived as holistic, integral, fun, and pleasurable?
	Value of music and care	Is there an ongoing financial investment in music care?
	Outbreaks/pandemic	Are outbreaks and pandemics used as opportunities to leverage music care?
	Support	Do you take advantage of coaching, training, and music care resources?
Staff—Who are the people most responsible for music care delivery?	Awareness of residents’ needs	Does staff use a person-centred approach to care?
	Buy-in	Is there buy-in for music care from most of the staff?
	Culture	Does the staff culture have a growth mindset?
	Number	Are there enough staff to enact a music care initiative?
	Knowledge of evaluation	Is there a demonstration of evidence-based practice?
	Reflective practice	Is there a demonstration of reflective practice?
	Community	Does the staff share a philosophy of LTC as the residents’ home and shared community?
	Adaptability	Can staff flex and adapt to a changing environment and resident needs?
Processes—What are the internal operational procedures impacting music care delivery?	Purposeful recruitment	Are you able to prioritize residents based on need?
	Planning and implementation	Are you able to create a detailed plan with sequential steps and put it into practice?
	Resources	Do you have the physical space, technology, and musical instruments needed for music care?
	Communication	Can you clearly articulate and mobilize your team around the plan?
	Tracking	Is there a procedure in place for tracking change?
	Time	Will time be allotted for learning music care practice?
	Construction	Is there scheduled site construction that could impact music care delivery?
Delivery—What makes music caredelivery work?	Flexibility	Can the music care initiative be adapted for optimization (i.e., language, group size)?
	Confidence	Do those delivering music care feel they can be confident and creative?
	Inclusivity	Can music care be used as a means of social bonding, especially amongst residents with different backgrounds i.e., ethnicity, socioeconomics?
	Frequency	How often can music care be delivered?
	Team approach	Is there a cohesive team approach for music care delivery?
	MCI appropriateness	What is the strategic fit of MCI within your context?
	Community partnerships	Are there community partners that might be willing to support music care practically?

## Data Availability

The data presented in this study are available on request from the corresponding author. The data are not publicly available due to privacy restrictions.

## Appendix A. The 10 Domains of Music Care

The music care delivery framework is comprised of ten domains, organized into three categories, that encompass situations where music is used in care. The domains are not meant to be rigid. Some music-based activities and strategies could be listed under more than one category or domain at a time.

**Category/Domain**	**Key Delivery Activity**	**Examples**
A. Intentional music-rich Environment		
Community	Accessing music performance between the healthcare site and community at large	School, community, faith groups, entertainers coming in, or residents attending symphony, fiddle club, or residents performing outside of setting
Musicking	Engaging informally and spontaneously with music	Playing instruments, singing, dancing
Programming	Integrating music formally in programs	Sing-along, listening groups, music bingo, music appreciation, Pathways Singing Program, Java Music Club
Technology	Incorporating technology to deliver music for a care-related goal	Playlists (Music & Memory, Playlist for Life protocols), bedside music terminals, virtual music instrument
Environmental Sound	Bringing intentionality to sounds made in the care environment	Recording of Tibetan bowls in prayer room, sounds to accompany labyrinth experience, Snoezelen rooms
B. Professionally Informed Music Care		
Music Therapy	Providing treatment using music within a therapeutic relationship as an accredited scope of practice	Client populations: mental health, rehabilitation, palliative, autism, etc.
Specialists	Performing therapeutically intended music by practitioners with certified training	Harp therapist, music thanatologist, bedside singers, healthcare musicians, participatory artists, music educators, community musicians, expressive art therapists
Music Medicine	Administering prescriptive music or sound-based interventions for medically related outcomes	Rhythmic auditory stimulation, vibroacoustic therapy, audioceuticals, melodic intonation therapy
C. Knowledge Development and Translation		
Training	Training to integrate music into regular care practice	Music Care Training, Neurologic Music Therapy Training, Music Therapy Con Ed, In-services, workshops
Research	Investing in evidence-based research using music and music strategies	Music and Health Research Collaboratory, McMaster Institute for Music and the Mind, Conrad Centre for Music Therapy, McGill Music and Perception Centre

## Appendix B. Memo of Understanding with LTC Home

Memorandum of Understanding

Between XXXXX LTC Home and Room 217 Foundation

This Memorandum of Understanding (MOU) sets forth the terms and understanding between XXXXX LTC Home and the Room 217 Foundation for the Music Care Partners Program.

Background: Music Care Partners is a six to eight-month project that explores how music can be integrated more fully into care to reduce the social isolation of residents in LTC homes. The partnership between XXXXX LTC Home and the Room 217 Foundation will be mutually beneficial. XXXXX LTC Home will gain expertise on the use of music in care and consultancy/support during the implementation and evaluation of a music care initiative. The Room 217 Foundation will gather quantitative and qualitative data to determine the effectiveness of the Partners Program at decreasing isolation and loneliness experienced by LTC residents.

Purpose: Through this partnership, music will be intentionally implemented at XXXXX to have positive impacts on LTC residents. Specifically, this project will:

- Reduce isolation and loneliness of marginalized residents;
- Increase engagement of all care partners with residents;
- Increase musical opportunities for connection and engagement for marginalized residents;
- Increase caregiver confidence using music 1:1 or in groups.

The above goals will be accomplished by undertaking the following activities:

XXXXXX LTC Home

- Provide one executive leader to work with Room 217 and lead the site team;
- Provide access to the RAI data coordinator to work with the Room 217 researcher;
- Enlist 10–24 staff and/or community members to participate in music care training, which will be delivered by Room 217; provide space for this two-day training;
- Create a site team of 10 staff and community members to be part of the Music Care Partners program. The leader of this team must have authority to make home-wide or home-area wide decisions about the implementation of the music care initiative;
- Host five 90-minute meetings (provide space and refreshments).

Room 217

- Provide music care training in-house at XXXXX;
- Provide a music care kit to XXXXX;
- Conduct participatory action research for the duration of the program;
- Collect resident outcome data related to self-perceived isolation and loneliness;
- Report the project outcomes to XXXXX (see below).

Reporting: The Room 217 Foundation will provide a comprehensive report at the culmination of the project, which will include statistical analysis of data collected, a summary of XXXXX LTC Home’s process, qualitative outcomes including resident stories, and a comprehensive guide explaining how to implement the Partners Program in-house without the support of Room 217. In addition, Room 217 will provide a large wall poster to showcase the process and results of the program.

Funding: The Partners Provincial Impact Program is funded by the Ontario Trillium Foundation. This MOU is not a commitment of funds between XXXXX and Room 217.

Duration: The estimated time frame for the Partners Program at XXXXX is xxxxxxxx to xxxxxxxx (approximately eight months.) This MOU is at-will and may be modified by mutual consent of authorized officials from XXXXX LTC Home and the Room 217 Foundation. This MOU shall become effective upon signature by the authorized officials from XXXXX LTC Home and the Room 217 Foundation and will remain in effect until modified or terminated by any one of the partners by mutual consent. In the absence of mutual agreement by the authorized officials from XXXXX and Room 217, this MOU shall end on xxxxxxx (agreed upon last day.)

Contact Information

XXXXX LTC Home

Representative:                Position:

Address:

Telephone:                  Email:

Room 217 Foundation

Chelsea Mackinnon, Research Lead

Box 145, Port Perry, ON

(905) 852-2499 cmackinnon@room217.ca

_ Date:

(Partner name, organization, position)

_ Date:

Chelsea Mackinnon, Room 217 Foundation, Research Lead

## Appendix C. Resident Informed Consent Letter to Participate

LETTER OF INFORMATION/CONSENT

Room 217 Foundation: Music Care Partners Program

Local Principal Investigator and Principal Investigator:

Chelsea Mackinnon, Education & Research Manager, Room 217, Primary Researcher

Maggie Li, Bachelor of Health Sciences, McMaster University, Researcher

Purpose: We are interested in learning how music can be integrated into daily life and experiences in the long-term care setting. The purpose of this study is to determine whether integrating music into daily life at your long-term care home can decrease isolation and loneliness experienced by residents. In other words, we want to know whether music initiatives benefit your health and/or overall wellbeing.

Procedure: You are invited to take part in this study by participating in the two-month music initiative at your long-term care home. You will be asked to complete one survey before beginning the program, and the same survey after completion of the two-month program. These surveys will be administered by a research assistant from McMaster University and will take approximately 10 minutes of your time.

Possible Risks: Risks from participating in the music initiative:

There are no significant physical risks from participating in the music initiative. While there may be some physical activity involved (such as tapping feet and hands, standing if able), it is very limited. If you feel uncomfortable or overwhelmed at any point during the music initiative, you can leave the room at any point. Music can also evoke strong emotions and memories. You can leave or stop at any time if you experience negative emotions or undesirable memory recall.

Risks from participating in the research study:

You may find completing the survey upsetting. In the event you become upset by any of the questions asked you can choose to skip those questions or stop the survey at any time. You may choose to resume at a later time or stop the project entirely. Healthcare providers from your long-term care home will be available to offer you support as needed.

Potential Benefits: We cannot promise any personal benefits to you from taking part in this study. However, potential benefits may include feelings of joy and happiness, and developing relationships with staff, community members, and/or other residents. Additionally, a benefit is that you will contribute to our understanding of the effects of using music at your long-term care home.

Confidentiality: Any information obtained through this study will be kept confidential. We will not use your name or any personal information that would allow you to be connected with your ratings on the scale. The results of this investigation will have no impact on the care that you receive. Once the study is complete, an archive of your survey form will be kept in a password-protected file on Chelsea Mackinnon’s computer. No personal information will be collected or recorded, which means that your results on the surveys will be kept confidential.

Participation and Withdrawal: Your participation in this study is voluntary. If you decide to be part of the study, you can stop at any time, even after signing the consent form or part way through the study. You can also decide to withdraw from the study but continue to attend the music initiative. If you decide to withdraw, it will not affect the care you receive at the long-term care home. You have the option of removing your data from the study. You may also refuse to answer any questions you don’t want to answer and still remain in the study.

Information about the Study Results: We expect to have the study results by May 2020. If you would like a brief summary of the results, please let me know how you would like it sent to you.

Questions about the Study: If you have questions or need more information about the study itself, please contact me at: cmackinnon@room217.ca & (905) 852-2499. This study has been reviewed by Veritas Independent Review Board (IRB). If you have any questions about your rights as a research participant or the Investigator’s responsibilities, you may contact the Manager of Veritas IRB 24 hours per day and seven days per week at 514-337-0442 or toll-free at 1-866-384-4221. An IRB is a group of scientific and non-scientific individuals who perform the initial and ongoing ethical review of the research study with the research participant’s rights and welfare in mind. If you have any study-related comments, complaints, or concerns, you should first contact the study investigator. Please call the IRB if you need to speak to a person independent from the Investigator and the research staff, and/or if the investigator and the research staff could not be reached.

Statement of Consent

I have read the information presented in the information letter about a study being conducted by Chelsea Mackinnon of the Room 217 Foundation. I have had the opportunity to ask questions about my involvement in this study and to receive additional details I requested. I understand that if I agree to participate in this study, I may withdraw from the study at any time. I have been given a signed copy of this form. I agree to participate in the study.

I would like to receive a summary of the study’s results. Yes No

If yes, where would you like the results sent:

Email or Address:

__________________________________________

Name of Participant (Printed)

Signature              Date

## Appendix D. Interview Questions

For Staff (either a site team leader, executive director, or staff not part of site team)

Length: no more than 15 min.

What are the barriers and enablers of music care implementation at the level of the:

1. Organization—including funding, leadership, board of directors, gaps, planning, sustainability, attitudes
2. Infrastructure—including material “stuff”, such as iPods, music care resources, instruments, staffing
3. Individual/person-level interactions—including interpersonal relationships, individual attitudes, individual relationships

For Residents

Length: 5 min

- How are you finding the music program/initiative/project (insert site-specific initiative name if appropriate)?
- Tell me a bit about your experience with music at (insert name) long-term care home.
